# Essential oil optimizes the susceptibility of *Callosobruchus maculatus* and enhances the nutritional qualities of stored cowpea *Vigna unguiculata*

**DOI:** 10.1098/rsos.170692

**Published:** 2017-08-23

**Authors:** Mazarin Akami, Hamada Chakira, Awawing A. Andongma, Kanjana Khaeso, Olajire A. Gbaye, Njintang Y. Nicolas, E.-N. Nukenine, Chang-Ying Niu

**Affiliations:** 1 Department of Biological Sciences, Faculty of Science, University of Ngaoundere, PO Box 454, Ngaoundere, Cameroon; 2 Department of Food Science and Nutrition, National School of Agro-Industrial Sciences, University of Ngaoundere, PO Box 454, Ngaoundere, Cameroon; 3 College of Plant Science and Technology, Huazhong Agricultural University, Wuhan 430070, China; 4 Department of Biology, Federal University of Technology, P.M.B. 704, Akure, Nigeria

**Keywords:** cowpea, *Callosobruchus maculatus*, *Lippia adoensis*, essential oil, nutritional parameters, anti-nutrients

## Abstract

The intensive use of synthetic pesticides in cowpea storage has led to the development of resistance by *Callosobruchus maculatus* and subsequent degradation of grain quality. In an attempt to circumvent these constraints, the susceptibility of *C. maculatus* to 2,2-dichlorovinyldimethyl phosphate (DDVP) and *Lippia adoensis* essential oil (EO) was investigated and variations in the proportions of nutritional values of treated grains 150 days after storage were assessed. The survival rate was recorded after five generations. The resistance index and biochemical parameters of grains were determined. The results from this study revealed that the survival rate and resistance index significantly increased proportionally with damage in DDVP treatments (*r* = 0.889; *p* = 0.018) while in EO treatments, those values remained low without significant variations (*p* = 0.0764) throughout the generations. DDVP stored grains yielded higher crude protein values, but lower carbohydrates, tannins, phenolics and minerals compared to EO. Eighteen amino acids were detected in EO treated grains and 14 in DDVP which was devoid of albumin and prolamin. *Lippia adoensis* EO could therefore represent a safe alternative bio-pesticide to cope with insect resistance and enhance the nutritional qualities of stored cowpea seeds.

## 1. Introduction

Cowpea *Vigna unguiculata* (L.) Walp. (Fabales: Fabaceae) is an economically important legume believed to have originated from Sub-Saharan Africa [1,2]. Cowpea is of paramount importance to the survival of low-income farmers in Africa and is consumed in many forms throughout the year [3]. It potentially contributes to poverty alleviation and reduction of food deficiency in Africa. In Cameroon, it is an important source of plant protein for humans and livestock [3,4] and contributes to soil fertility [5–7]. Furthermore, cowpea is well adapted to the stressful climatic conditions of tropical Africa as it can tolerate drought and warm weather, and could be grown in tropical and sub-tropical regions [8,9]. Approximately 50–80% of cowpea grains are lost during the storage stage due to pest insect attacks [4]. The far-north region of Cameroon produces about 80% of the country's cowpea [4]. However, the floods that occurred in 2012 destroyed large farmlands leading to a decrease in its production [10,11]. This, coupled with Boko Haram terrorist attacks and the immigration of refugees from neighbouring Nigeria and Central African Republic [10], has led to an increase in cowpea demand. Therefore, there is a need to boost cowpea production and limit its post-harvest losses.

*Callosobruchus maculatus* F. (Coleoptera: Chrysomelidae) is a field to store pest of cowpea as its infestation starts on the dried mature pods in the field [12]. It causes serious qualitative and quantitative damage to the grains [13–15]. The adult is harmless as it does not feed, but lays eggs on the grains which later hatch and penetrate into the seed cotyledons [8], where the larvae and pupae develop and could completely damage seed viability and nutritional quality [16–18]. Chemical pesticides are mainly used to minimize insect damage during storage [19–21]. The misuse of these pesticides has led to environmental hazards, health concerns and the development of insect resistance [22–24].

In order to ensure food security and overcome malnutrition, hunger and poverty in Sub-Saharan Africa, it is of utmost importance to enhance cowpea production to meet the needs of the rising African population [10]. Cost-effective control strategies that could efficiently limit the damage caused by *C. maculatus* to cowpea and enhance the nutritional and market value of the stored grains are the way forward to cope with these challenges. Several studies have been carried out to understand the beneficial effects of plant materials in protecting cowpea against *C. maculatus* infestation and their ability to reduce the resistance of this pest [4,13,23,25]. However, many food safety related questions still need to be addressed as far as the storage is concerned. For example, what is the long-term nutritional cost and benefit of storing cowpea with synthetic insecticides such as 2,2-dichlorovinyldimethyl phosphate (DDVP) and plant essential oils (EO)? Could *Lippia adoensis* EO be used as a supplement to optimize the nutritional capacity of grains? Conventional methods have been used for physiological and biochemical analysis. The data have been processed and the results are discussed.

## 2. Material and methods

### 2.1. Plant materials and insects preparations

#### 2.1.1. Cowpea seed collection

Twenty kilograms of dried un-infested cowpea seeds (black eye beans) of the Mozongo variety were collected from Lara in the far-north region of Cameroon (28°26′67.18′′ S, 38°43′57.52′′ E). Seed preparations were carried out as previously described [8]. The seeds were cleaned and disinfested by storing them at −20°C for 10 days. After disinfestation, the seeds were left undisturbed in the laboratory for 5 days under the following conditions: 28 ± 5°C, 83 ± 5% RH and L : D 10 : 14 to avoid mouldiness.

#### 2.1.2. Insect rearing techniques

The laboratory populations of *C. maculatus* were established from infested cowpea seeds from Lara, Cameroon and reared in the laboratory for 4 years. Similarly, wild populations used for this study were collected from Lara. About 100 g of disinfested cowpea was introduced in transparent glass jars and reared until adult emergence of the first progeny 26 ± 3 days later. Rearing was carried out in the insect laboratory at the University of Ngaoundere.

#### 2.1.3. Preparation of essential oil

Fifty grams of fresh *L. adoensis* leaves were harvested around University of Ngaoundere (Cameroon), shade-dried for one week and ground. The resulting powder was subjected to hydro-distillation using a modified Clevenger-type apparatus (XWD-C-1000, Shanghai XinWangDe Laboratory Equipment Co., China) for 6 h and the oil was extracted with *n*-hexane. Anhydrous sodium sulfate was used to dry excess water after extraction [26–28]. The recovered crude EO was stored in an airtight glass container and refrigerated at 4°C.

#### 2.1.4. Pesticide preparations

The DDVP (PESTANAL®, Analytical Standard, C_4_H_7_Cl_2_O_4_P) used in this study was obtained from Sigma-Aldrich (ID: 45441, molecular weight: 220.98; CAS number: 62-73-7) and the EO was extracted as described above. The preparation of DDVP was carried out according to the manufacturer instructions. Briefly, various concentrations were dissolved in a solvent (dimethylsulfoxide, DMSO) which acts as emulsifier. Stock solutions of 10 000 ppm each were made. Three gradual concentrations 2 g µl^−1^, 5 g µl^−1^ and 10 g µl^−1^ representing 0.0001%, 0.005% and 0.01% of DDVP [23] or 0.25 ppm, 0.5 ppm and 1 ppm were pipetted for bioassays. DMSO (0.0%) served as control treatments.

### 2.2. Experimental procedures

#### 2.2.1. Fitness measurements over generations

##### 2.2.1.1. Mortality rates

The protocol described by Oyeniyi *et al.* [18] was adopted with slight modifications. Fifty grams of cowpea seeds was transferred into ten glass containers with a diameter of 9 cm and thoroughly mixed with 1 ml of DMSO (control) and 2 g µl^−1^, 5 g µl^−1^ and 10 g µl^−1^ of DDVP and EO, respectively. After mixing, the solvent was allowed to evaporate for twenty minutes. Ten pairs of male and female (1–2 days old) of *C. maculatus* were introduced into the different treatment containers and covered with a mesh lid. Four replications were set for each treatment. The adult mortality was assessed at 1, 2, 4 and 6 days after infestations. Bruchids were considered dead when they showed no response after their abdomens were gently prodded with a brush [8,18].

##### 2.2.1.2. Pupal eclosion rates

All insects were removed after 6 days post-treatment and the dead ones were discarded. The surviving insects were re-introduced into the initial treatment containers and were allowed to lay eggs for 5–6 days after infestation. The number of eggs on each seed was counted under a light microscope and constituted the potential number of adults expected to emerge in subsequent generation [8]. At the emergence, a daily count was made and newly emerged insects were progressively removed to avoid interference with the next generation [29]. The newly emerged adults were later introduced into a new container (if the container is on one) or into new containers (if the containers were more than one) before the next experiment. The percentage of adult emergence in each container was evaluated as the difference of the number of eggs laid and the resulting adults that emerged.

#### 2.2.2. Proximate analysis

##### 2.2.2.1. Experimental design

The nutritional quality of the grains exposed to the different treatments was evaluated at the end of the storage period (150 days). The negative control (infested, hereinafter) was only treated with the solvent and infested with 10 couples of adult *C. maculatus*. Non-treated and non-infested cowpea seeds formed the positive control (standard, hereinafter). The standard constitutes the reference cowpea seeds from which the nutritional parameters were evaluated at the beginning of the storage. The tests were carried out under the same thermo-hygrometric and photoperiodic experimental conditions as described above. Four replications were set up for each treatment group.

##### 2.2.2.2. Sample preparation

After 150 days of storage, the treated cowpea seeds from all the treatments (DDVP, EO, standard, and infested) were finely ground into flour using a Knifetec 1095 Sample Mill (Foss Tecato, Hoganas, Sweden). The powder was sieved using a 70 mesh screen, transferred into airtight polythene bags and stored at −20°C from which 15 g were used for subsequent analysis. All the reagents used in this study were of analytical grade and stored according to manufacturer's instructions.

##### 2.2.2.3. Determination of proximate composition of seeds

The cowpea powder was assessed for crude protein, fat, ash, moisture, calcium, magnesium, potassium, phosphorus, zinc, copper, iron, manganese, selenium, sodium, sulfur, boron, and cobalt using classical procedures [30–32]. Briefly, the crude fat was extracted by Soxhlet extraction with diethyl ether; crude ash by incineration in a carbolite furnace at 600°C for 8 h and cooled at room temperature; crude protein by the Kjeldahl method [33], quantified and calculated as N × 6.25; the moisture gravimetrically; and dietary fibre by a chemical-gravimetric method with fibre analyser ANCOM 220 (ANKOM Technology, USA) and the means reported on dry weight basis [34–37]. The carbohydrate was estimated by Anthrone's method [38,39]. Full details are provided in the supporting materials.

##### 2.2.2.4. Determination of protein fractions

Different protein fractions albumins (water-soluble), globulins (salt-soluble), prolamins (alcohol-soluble) and glutelins (alkali-soluble) were extracted sequentially based on their solubility criteria in respective solvents (distilled water, 1% sodium chloride, 60% ethanol, and 0.4% sodium hydroxide) [40,41]. The proportion of each soluble fraction obtained was expressed on the basis of total soluble protein [42,43].

##### 2.2.2.5. Determination of amino acid profile

Crude protein from each treatment was separately dried to constant weight, defatted, hydrolysed with 6 M HCl at 100°C for 24 h under vacuum and evaporated. The residue was loaded into a Technicon sequential Multi-Sample Amino Acid Analyzer (TSM, model DNA O2O9) that aims at designing, separating, detecting and quantifying free, acidic, neutral and basic amino acids of the hydrolysate [44,45]. The protein quality was estimated by determining the total amino acids (TAA), the essential amino acids (EAA), the chemical score (CS), the essential amino acids index (EAAI), and the net protein value (NPV) as previously reported [46]. The contents of different amino acids were presented as a percentage of the content of the same amino acid in the standard protein and expressed as g/16 g of nitrogen to the equivalent of g/100 g protein on dry weight basis [47–49]. Scoring pattern for schoolchildren (10–12 years old) was used as reference pattern for the comparison of amino acids in our samples [50,51] (electronic supplementary material, table S1).

##### 2.2.2.6. Determination of total phenolics

The total phenolics were measured and expressed as mg gallic acid equivalents (GAE)/g of extract through the calibration of standard curve of gallic acid (0–100 mg ml^−1^ range) (Sigma, USA) [52–54]. For the procedure, 100 mg of each flour was weighted and taken in test tubes. One millilitre of deionized distilled water and Folin-Ciocalteau phenol reagent was added into the test tubes at equal proportion (1 : 1). Then 2.5 ml of sodium carbonate solution (30% w/v) was added sequentially in each test tube and the mixture was vortexed before being placed in the dark for 40 min. The absorbance was read at 725 nm using a spectrophotometer (Eppendorf AG, Germany) against the reagent blank [7,55].

#### 2.2.3. Determination of condensed tannins

The condensed tannin content was estimated by incubating sample extracts with polyvinyl polypyrrolidone (PVPP). One hundred milligrams of PVPP was weighed and transferred into test tubes and mixed with an equal volume (1 : 1) ml of deionized distilled water and cowpea flour. The tubes were vortexed and left unattended at 4°C for 30 min. Soon after, the tubes were vortexed again and centrifuged at 5000 rpm for 5 min. The absorbance of the supernatant was measured at 725 nm using spectrophotometer (Eppendorf AG, Germany).

### 2.3. Data processing

Abbott [56] formula was used to correct adult mortality data. Thereafter, corrected data on mortality counts and those obtained from biochemical analysis and survivorship were arcsine-transformed and the cumulative number of progenies was log-transformed. The transformed data were later subjected to one way ANOVA at probability of 0.05 and mean separation was done using Duncan's multiple range test, where significance difference existed. The concentration of DDVP and EO needed to evoke 50% (LC_50_) and 95% (LC_95_) mortality of *C. maculatus* at every generation after treatment application was determined using probit analysis [57]. All analyses were carried out using Statistical Package for Social Sciences (SPSS) 20.0 software package. Graphs were constructed using OriginPro version 8.5.1. Based on the lethal concentrations (LC), the resistance index (RI) of each generation was calculated as previously described [23] with modifications. Calculation formulae of CS, EAAI, NPV, biological value (BV) and predicting protein efficiency ratio (P-PER) are provided in the electronic supplementary material. The nitrogen-free extract (NFE) was estimated as follows: NFE= 100 − (% Moisture + % Crude protein + % Fat + % Ash + % Crude fibre). The tannin content of the sample was calculated according to the equation: Tannin = Total phenolics − Non-tannin phenolics [58,59].

### 2.4. Permission to carry out fieldwork

All the experiments were carried out in the laboratory and the sample collections were done in the experimental field of the University of Ngaoundere. We therefore did not require any fieldwork permissions or licences.

## 3. Results

### 3.1. Susceptibility of *C. maculatus* to EO and DDVP over five generations

#### 3.1.1. Variations in adult mortality

**Figure 1.**
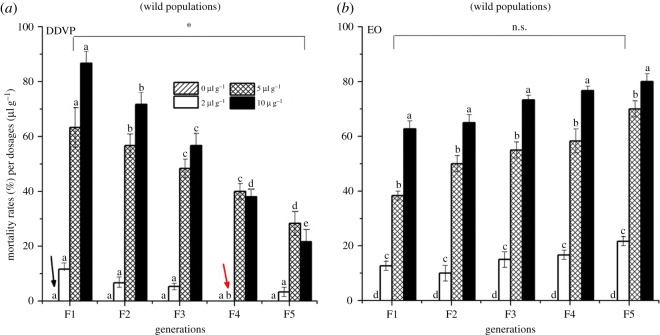
Adult mortality of *Callosobruchus maculatus* wild populations recorded over generations (F1 to F5) exposed to four different concentrations of DDVP (*a*) and EO (*b*). Means with different letters between generations are significantly different after comparison with Duncan's test at *p* = 0.05; *ns*, not significant (*p* = 0.0764); highly significantly different (*p = 0.0176).

The responses of wild populations of *C. maculatus* to EO and DDVP varied with increasing concentrations and generations. The adult mortality increased proportionally with the concentrations of DDVP within generations but decreased between generations (*r *= −0879; *p* = 0.0287) except in control groups (figure 1, black arrow). Meanwhile, the susceptibility of *C. maculatus* to EO remained likely constant throughout generations (*r* = 0.296; *p* = 0.0532) (figure 1, EO) although slight increase over generation was recorded. The drastic reduction in adult mortality from the third generation indicates an increasing tolerance of bruchids to DDVP (*p* = 0.018) (figure 1, DDVP). However, on the basis of the results, the laboratory populations were the most susceptible to both pesticides (electronic supplementary material, figure S1).

#### 3.1.2. Resistance parameters

The survival rate (SR) and resistance index (RI_50_ and RI_95_) of DDVP wild populations of *C. maculatus* increased significantly from the third generation (ranging from 46.11% to 95% for SR, from 3.52 to 22 for RI_50_ and from 12.8 to 20.20 for RI_95_) (table 1) compared to the standard laboratory populations. However, these parameters remained lower and did not vary significantly in EO (*p* = 0.0764) over generations (table 1). The results showed that the toxicity of DDVP was inversely proportional to generations whereas in EO, it remained generally constant over generations (figure 1; table 1).

**Table 1.** Linear regression of lethal parameters of DDVP and EO to adult *Callosobruchus maculatus* over generations. DDVP: 2,2-dichlorovinyldimethyl phosphate; EO: essential oil; P: products; G: generations; *χ*^2^: chi-square; s.e.: standard error; FL: fiducial limits; LC_50_ and LC_95_: lethal concentration killing 50 and 95% of *C. maculatus* adults, respectively; RI: resistance index; values followed by different letter(s) within columns are significantly different; *SR_g_*: survival rate per generation.

P	G	slope (±s.e.)	*χ*^2^	LC_50_ (50% FL)	LC_95_ (95% FL)	RI_50_	RI_95_	*SR_g_* (%)
DDVP	F1	0.87 ± 0.15	36.37^e^	0.020 (0.01–0.03)	0.87 (0.06–1.02)	2.56^e^	6.30^e^	46.11^e^
	F2	0.79 ± 0.10	67.34^d^	0.021 (0.01–0.04)	1.24 (1.19–1.74)	2.38^d^	8.99^d^	55.00^d^
	F3	0.98 ± 0.10	27.18^c^	0.032 (0.02–0.05)	1.76 (1.67–2.15)	3.52^c^	12.8^c^	77.22^c^
	F4	0.84 ± 0.11	80.5^b^	0.102 (0.09–0.16)	1.96 (1.73–2.13)	11.33^b^	14.2^b^	86.67^b^
	F5	0.94 ± 0.12	75.9^a^	0.198 (0.28–0.22)	2.78 (2.74–3.24)	22.0^a^	20.2^a^	95.0^a^
	standard	1.08 ± 0.009	96.20	0.009 (0.007–0.018)	0.14 (0.12–0.16)	1.00	1.00	97.17^a^
EO	F1	0.71 ± 0.13	17.63^d^	0.012 (0.01–0.02)	0.044 (0.04–0.08)	2.4^a^	2.59^a^	66.67^c^
	F2	0.79 ± 0.12	58.10^c^	0.016 (0.012–0.023)	0.038 (0.04–0.05)	3.2^a^	2.23^a^	58.33^b^
	F3	0.97 ± 0.10	62.14^b^	0.017 (0.014–0.19)	0.059 (0.05–0.08)	3.4^a^	3.47^a^	52.22^b^
	F4	0.89 ± 0.11	61.64^b^	0.015 (0.013–0.021)	0.073 (0.07–0.08)	3.0^a^	4.29^a^	47.78^b^
	F5	1.09 ± 0.12	72.84^a^	0.018 (0.014–0.025)	1.020 (0.99–1.26)	3.6^a^	3.82^a^	42.78^b^
	standard	0.99 ± 0.11	97.12	0.005 (0.003–0.01)	0.017 (0.013–0.023)	1.00	1.00	96.89^a^

### 3.2. Variations in the nutritional parameters of cowpea seeds

#### 3.2.1. Chemical variations of constituents

**Figure 2.**
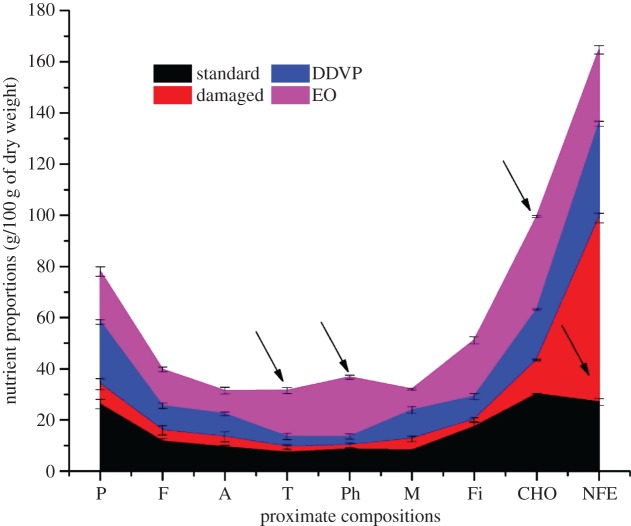
Variation of the nutritional parameters of cowpea seeds after 150 days of storage with 10 g µl^−1^ DDVP (2,2-dichlorovinyldimethyl phosphate) and EO (essential oil). P, protein; F, fat, A, ash; T, tannins; Ph, phenolics; M, moisture; Fi, fibre; NFE, nitrogen-free extract; CHO, carbohydrates. Each horizontal lane shown in different colours represents the four different seed groups analysed and each proximate value represents the mean ± standard error of four replications.

**Figure 3.**
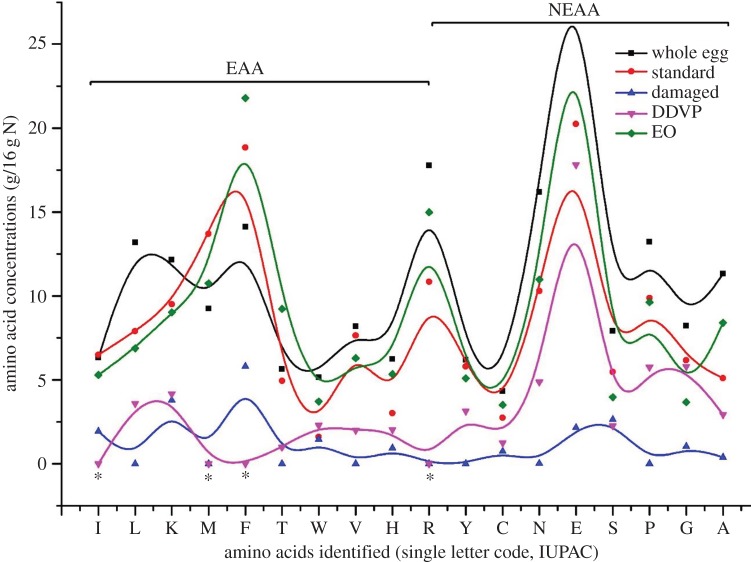
Variations of amino acid profiles (g/16 g of nitrogen or g/100 g of protein) of proteins from cowpea grains after 150 days storage with 10 g µl^−1^ of DDVP and EO compared to the whole egg reference protein, non-infested (standard) and damaged non-infested (standard) and damaged. EAA, essential amino acids; NEAA, non-essential amino acids; DDVP, 2,2-dichlorovinyldimethyl phosphate; EO, essential oil; I, isoleucine; L, leucine; K, lysine; M, methionine; F, phenylalanine; T, threonine; W, tryptophane; V, valine; H, histidine; R, arginine; Y, tyrosine; C, cysteine; N, asparagine; E, glutamic acid; S, serine; P, proline; G, glycine; A, alanine.

The damage of the infested grains significantly altered almost all the nutritional parameters of grains. The nutritional compounds were higher in EO treatments compared with the control (18.40 ± 1.17, 23.24 ± 0.78 and 36.29 ± 0.43 g/100 g dry weight (dw), respectively) (figure 3, up-ray arrows). However, the crude protein content in DDVP treatments was higher than in EO (26.18 ± 0.92 and 19.74 ± 1.88 g/100 g dw, respectively) (figure 2). The DDVP and the infested grains both recorded a significant reduction (*p *= 0.02213) in fat, ash, fibre, phenolic and tannin contents whereas the moisture content in DDVP treatments generally compared with the standard treatments (figure 2).

#### 3.2.2. Mineral variations

Thirteen micro- and macro-minerals were identified (table 2). Calcium and potassium were the most dominant macro-elements in all treatments. They were more found in EO treated cowpea with about 35.17% increase compared with the standard (table 3) whereas in DDVP treatments they decreased by 65.02%. The micro-elements iron and selenium were highly represented in the standard but their amounts significantly decreased in DDVP treatments.

**Table 2.** Variations in the proportion of minerals and salts (mean ± s.e.) of cowpea seeds treated with 10 g µl^−1^ of DDVP and EO after 150 days of storage. Each value represents the mean of four replications ± s.e. Means followed by the same letter(s) in a column are not significantly different after comparison with Duncan's test at *p* = 0.05; DDVP: 2,2-dichlorovinyldimethyl phosphate; EO: essential oil; s.e.: standard error; values within rows followed in brackets by: *ns*: not significant (*p* < 0. 05) with Duncan's multiple range test; *: slightly significant; **: significant (*p* < 0.01); ***: highly significant (*p* < 0.001) compared to the standard.

minerals (mg/100 g)	standard	damaged	DDVP	EO
macro-elements				
calcium	4.87 ± 0.002^d^	0.974 ± 0.004^c^(*)	1.27 ± 0.03^a^(*)	7.74 ± 0.24^b^(**)
phosphorus	2.26 ± 0.007^a^	0.001 ± 0.014^b^(***)	0.24 ± 0.009^b^(**)	3.58 ± 0.45^c^(ns)
magnesium	2.57 ± 0.009^a^	0.001 ± 0.018^a^(***)	0.51 ± 0.004^c^(**)	1.27 ± 0.01^a^(*)
potassium	3.63 ± 0.002^c^	0.726 ± 0.004^c^(***)	1.68 ± 0.092^a^(*)	7.26 ± 0.03^b^(***)
sodium	2.20 ± 0.001^b^	0.440 ± 0.003^b^(*)	0.37 ± 0.008^b^(^*)^	1.09 ± 0.09^a^(^**)^
sulfur	0.77 ± 0.139^a^	0.001 ± 0.278^a^(***)	1.63 ± 0.002^a^(**)	1.09 ± 0.00^a^(ns)
micro-elements				
zinc	34.03 ± 2.98^f^	6.81 ± 5.96^f^(***)	9.09 ± 1.79^e^(***)	28.31 ± 1.86^d^(*)
copper	10.34 ± 1.33^e^	1.07 ± 2.66^e^(***)	11.32 ± 1.55^e^^(ns)^	13.38 ± 0.99^e^^(ns)^
manganese	78.93 ± 2.17^a^	15.79 ± 4.34^e,b^(**)	16.27 ± 1.99^d^(**)	34.13 ± 2.01^d^(*)
iron	450.29 ± 32.29^d^	76.02 ± 64.58^d^(***)	138.22 ± 2.67^c^^(ns)^	371.38 ± 17.24^c^(*)
selenium	296.89 ± 2.47^c^	59.38 ± 4.94^c^(***)	311.43 ± 1.58^c^^(ns)^	184.19 ± 3.29^c^(*)
boron	58.83 ± 3.43^b^	11.78 ± 6.86^b^(***)	73.81 ± 1.09^b^(**)	60.17 ± 3.18^b^^(ns)^
cobalt	84.41 ± 2.83^a^	16.89 ± 5.66^a^(***)	95.49 ± 2.08^a(ns)^	107.07 ± 4.17^a^(**)

**Table 3.** Protein fractions (g/100 g of protein) of cowpea grains after 150 days of storage with DDVP and EO. (—): not found.

	standard	damaged	DDVP	EO
protein (%)	26.18	7.82	24.29	19.74
fractions				
albumin	7.14	—	—	6.19
globulin	15.74	2.04	7.09	17.81
glutelin	5.01	—	2.14	7.18
prolamin	2.72	0.03	—	3.19

#### 3.2.3. Variations of amino acid compositions over the storage period

The variation in the general amino acid compositions of cowpea after 150 days of storage with DDVP and EO is presented in figure 3. A total of 18 amino acids were recorded in EO and non-treated grains and 14 in DDVP treatments. The amino acids isoleucine, methionine, phenylalanine, arginine and protein fractions albumin and prolamin were missing in DDVP treatments (table 3, figure 3). The concentrations of six amino acids (phenylalanine, threonine, tryptophan, histidine, arginine and glutamate) significantly increased (*p* = 0.028) in EO treated cowpea seeds alongside with globulin and glutelin compared with the standard cowpea. Numerical amounts of different amino acids and the FAO/WHO [50] reference patterns are provided in table S1 of the electronic supplementary material.

#### 3.2.4. Variations in protein fractions

Protein fractions were estimated and huge variations were observed under different storage conditions. Globulin was the most abundant fraction recorded in all the treatments, followed by glutelin in standard and EO. Albumin and glutelin were not detected in the damaged cowpea seeds whereas in the DDVP treated seeds, the missing fractions were albumin and prolamin (table 3).

### 3.3. Evaluation of protein quality coefficients of cowpea seeds

Amino acid score was based on the amount of the first limiting amino acid, and its calculation included the use of FAO/WHO/UNU [50] protein requirement pattern. The variation of the nutritional parameters in the four treatments is presented in table 4. Proteins derived from EO treatments had the best quality and higher nutritional values (table 4).

**Table 4.** Quality assessment of proteins extracted from different cowpea seeds after 150 days of storage with DDVP and EO treatments. TAA: total amino acid; EAAI: essential amino acid index; TSAA: total sulfur amino acids; TBAA: total basic amino acid; NPV: net protein value; CS: chemical score; BV: biological values; P-PER: predicted protein efficiency ratio.

parameters	whole egg	standard	damaged	DDVP	EO
TAA (%)	100	75.82	10.54	29.69	84.71
TSAA (%)	100	121.31	5.46	9.14	105.16
TBAA (%)	100	64.58	13.02	17.11	81.14
EAAI	100	84.39	14.59	16.49	96.33
NPV	39.83	8.15	0.12	4.21	10.30
CS (%)	100	31.13	0.15	17.35	52.20
BV	103.32	87.90	7.44	8.72	97.96
P-PER	4.87	2.51	−0.47	0.83	2.12

## 4. Discussion

The results from this study revealed that the susceptibility of *C. maculatus* to the pesticides varied with their types, concentrations and generations of the insects. The significant decrease in the mortality, fecundity and pupal eclosion rates over generations in wild populations of bruchids exposed to DDVP indicates their ability to adapt and tolerate different concentrations of DDVP from one generation to another, thus leading to the development of resistance. However, no significant variations in the mortality were observed in EO treatments except the number of eggs and the resulting adults that slightly decreased.

The established laboratory populations were the most susceptible with the highest mortality in all treatments. This could be because they have never been exposed to DDVP and therefore have not developed resistance to this insecticide (electronic supplementary material, figure S1). The cowpea seeds were collected from a locality where DDVP is intensively being used by farmers before bagging the cowpea seeds for storage. Unfortunately, repeated misuse and over-dosage have led to the development of resistance which was progressively acquired with the exposure to DDVP over several generations. Previous studies have reported the acquisition of resistance to synthetic insecticides by some pest insects in storage. For example, *Sitophilus* spp., *Tribolium castaneum* (Herbst) (Coleloptera: Tenebrionidae) and *Rhyzopertha dominica* (F.) (Coleoptera: Bostrichidae) were shown to be resistant to malathion and methyl bromide [60,61]. In the same light, several authors attributed the resistance of pests to several parameters such as the thickness of insect exoskeleton, type and concentration of insecticide, the ability of the insect to metabolize a poisonous substance, the type of grains, and the insect location [62–64].

The sustainable management of pests does not aim at eradicating them but to maintain their population under a noxious threshold [65]. Therefore, finding a pesticide that could meet this requirement stands as a great hope toward the development of an ecofriendly, targeted, efficient and sustainable pesticide. The result obtained from EO is a clear indication of the failure of the insect to develop any remarkable tolerance across the generations. This may be due to the multiple active sites and lipophilic properties of EO constituents that impaired the insect physiology and thereby not enabling it to initiate any resistance [4,66,67]. This eventually led to the disruption of the normal copulatory activities as reported by Oni & Ileke [68] and Adedire *et al*. [69]. Most of the eggs laid could not complete their life cycle thereby leading to a reduced adult population to form the subsequent generations and limiting the seed damage. EO available secondary metabolites might have subtly altered detoxification and nutrition processes and regulated better the frequency of emergence of new adults and maintained less harmful insect population.

One of the major challenging steps of cowpea production remains the storage of harvested crops in such a way that it meets the nutritional requirements of consumers and livestock and sustains good marketing values [65]. Therefore, appropriate protection measures may play an important role toward solving this challenge. According to the FAO report on handling and storage of food grains in tropical and subtropical areas as reported by Hou *et al*. [70], the protection against pests could only be efficient if it helps improving the local processing methods to retain inherent natural nutritional value of produce.

The highest coefficients of the nutritional values recorded in EO treated cowpea seeds compared with DDVP treated and standard is a clear indication of its capacity to boost the nutritional compositions of the grains to better meet the demands in protein and other key nutrients. The nutritional constituents (proteins, fats, carbohydrates) recorded in EO treatments were higher than most of those previously reported in cowpea [55]. However, Gupta *et al*. [71] observed higher content (27.7 ± 0.22 g/100 g) of protein from COVU-702 cowpea genotype than that of our study. The PER-R, the CS, the BV and EAAI were all higher than those recorded in DDVP treatments. EAAI (96.33) and BV (89.99) of EO treated grains were higher than those reported by Ilesanmi & Gungula [46] when they use neem (*Azadirachta indica*) and moringa (*Moringa oleifera*) seed oils. From the predicted protein efficiency ratio, essential amino acids index and biological value alongside with their protein fractions globulin, albumin, glutelin and prolamin most of which values increased, it could be inferred that preserving cowpea with EO is a safe and effective way to protect the grains against insect attack and optimize their protein quality [72]. Amino acids remain a prerequisite for the synthesis of proteins and other important nitrogen-containing compounds [47]. A diet containing an optimal amount of amino acids will ensure a good physiological functioning of the body [10].

EO treated cowpea could be used to enhance and maintain a constant secretion of haemoglobin and red blood cells [10]. Other minerals like calcium, manganese, phosphorus, zinc, magnesium, potassium, copper, cobalt, boron and selenium may also help in fuelling enzymatic reactions, maintaining strong bones in addition to ensuring good functioning of muscles [10].

The anti-nutritional compounds (tannins, phenols) varied in all the treatments but most importantly, they were significantly higher in EO treated grains. The highest protection observed in EO groups could be attributed to these anti-nutritional constituents whose astringent taste affects the palatability of grains, disrupts feeding behaviours [73], and reinforces their resistance to insect infestations [74]. In the same light, the phenolics from seeds are also doubly beneficial for the seeds themselves as well as for consumers because a phenolic enriched diet helps to prevent degenerative diseases and extend the lifespan in humans [75–77].

## 5. Conclusion

Based on our results, the impact of the infestation has reduced the nutritional quality of the commodity as a huge difference between unaffected (standard) and affected (damaged) was observed in all the measured parameters. The damaged seeds lost both nutritional quality and virtual market acceptance. It is therefore a threat to food security, as farmers will even lose the interest to produce such a crop. The use of DDVP, although effective at adult stage, induces the development of resistance in *C. maculatus* and contributes to the destruction of most of the key nutrients of the grains. This renders the seeds unsuitable for human consumption. On the contrary, *Lippia adoensis* EO maintained appropriate and suitable proportions of most of the essential amino acids to fulfil human nutritional requirements. It can therefore be incorporated in the storage system of cowpea in Sub-Saharan Africa and Cameroon in particular. Further investigations are needed for cost-effective evaluations of *Lippia adoensis* EO in stored cowpea seeds.

## Supplementary Material

Electronic supplementary materials (ESM 1)

## Supplementary Material

Electronic supplementary materials (ESM 2)

## Data accessibility

All datasets supporting this article have been uploaded as part of the electronic supplementary material and deposited at Dryad (http://dx.doi.org/10.5061/dryad.3rt12) [78] and Open Science Frame (https://osf.io/y6w38/).
