# Supplementary material for: Essential oil optimizes the susceptibility of Callosobruchus maculatus and enhances the nutritional qualities of stored cowpea Vigna unguiculata
Source: R Soc Open Sci. 2017 Aug 23;4(8):170692. doi: 10.1098/rsos.170692 (PMC5579128; doi:10.1098/rsos.170692)
Supplement: Electronic supplementary materials (ESM 1) [file rsos170692supp1.pdf]

Electronic supplementary materials (ESM 1): Additional results and FAO/WHO/ONU (1985) protein reference pattern

Essential oil optimizes the susceptibility of *Callosobruchus maculatus* and enhances the nutritional qualities of stored cowpea *Vigna unguiculata*

Mazarin Akami[1,2*], Hamada Chakira[2], Awawing A. Andongma[2], Kanjana Khaeso[2], Olajire A. Gbaye[3], Njintang Y. Nicolas[1,4], E-N Nukenine[1], and Chang-Ying Niu[2*]

1. Mortality rates in laboratory populations of *C. maculatus*

Figure S1 presents the mortality of *C. macultus* laboratory strains to EO and DDVP. Their susceptibility remained higher throughout progenies. It remained likely constant and slight decrease in the mortality from the 4th and 5th generation in both pesticides (red circle). This suggests that the laboratory strains started acquiring resistance later than the wild strains which started from the 2nd generation. No significant decrease in the mortality was recorded in both cases (p=0.059 and p=0.078) except in control groups (0 g/µl).

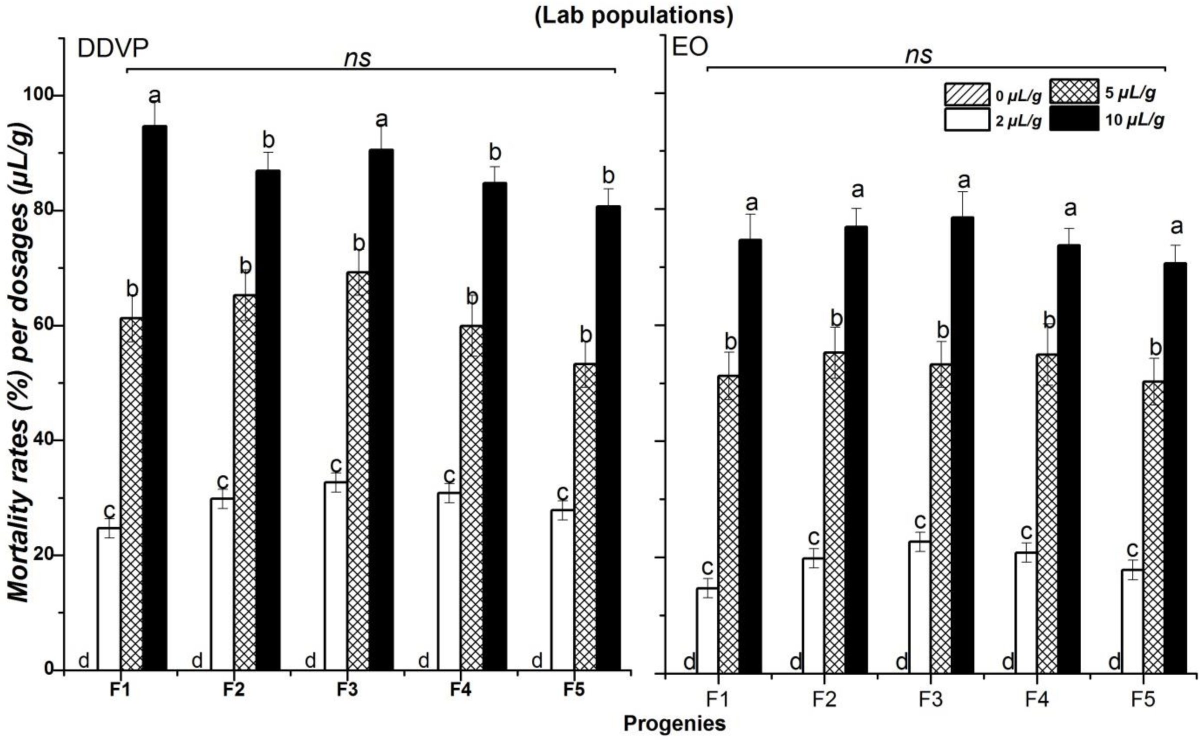

Figure S1. Adult mortality of *Callosobruchus maculatus* laboratory strains recorded over

generations (F1 to F5) exposed to four different concentrations (g/µl) of DDVP and EO. Means with different letters within generation are significantly different and means with the same letter(s) between generations are not significantly different after comparison with Duncan's Test at p= 0.05.

2. FAO/WHO/ONU (1985) protein reference pattern

Table S1. Variation in the proportions of Amino acids (g/100g of protein) of cowpea grains after 150 days of storage with 10g/µL of **DDVP** and **EO.**

	Code	Amino Acids	Whole Egg	STD	DMD	DDVP	EO	FAO/WHO/ONU (1985)* (mg)
	I	Isoleucine	7.32	6.49	1.93	-	5.29	37
	L	Leucine	13.18	7.9	-	3.58	6.88	56
E	K	Lysine	12.15	9.5	3.78	4.17	9.03	75
A	M	Methionine	9.24	13.7	-	-	10.75	34(M+C)
A	F	Phenylalanine	14.11	18.84	5.81	-	21.79	34(F+Y)
	T	Threonine	5.65	4.94	-	**0.98**	9.23	44
	W	Tryptophane	**5.14**	**1.6**	1.45	2.29	3.71	4.6
	V	Valine	8.18	7.64	-	1.99	6.29	41
	H	Histidine	6.24	3.017	0.93	2.02	5.34	
	R	Arginine	17.78	10.84	-	-	14.98	
	Y	Tyrosine	6.19	5.8	-	3.13	**5.09**	
	C	Cysteine	4.32	2.75	0.74	1.24	3.51	
N	N	Aspargine	16.19	10.29	0.024	4.87	10.97	
E	E	Glutamate	32.74	20.24	2.16	17.8	29.27	
A	S	Serine	7.91	5.47	2.64	2.26	3.97	
A	P	Proline	13.22	9.88	-	5.76	9.63	
	G	Glycine	8.21	6.17	**1.03**	5.78	3.67	
	A	Alanine	11.31	5.11	0.39	2.93	8.4	
		**Protein (%)**	**39.83**	**26.18**	**7.82**	**24.29**	**19.74**	

EAA: Essential Amino Acids; NEAA: Non-Essential Amino Acids; STD: Standard; DMD: Damaged; WHO: World Health Organization; FAO: Food and Agriculture Organization; (-): not found. * The 1989 Consultation recognized that the amino acid scoring pattern proposed in 1985 for children of preschool age was the most suitable pattern for use in the evaluation of dietary protein quality for all age groups, except infants FAO/WHO/ONU [1]. These values of essential amino acids were suggested by FAO/WHO/ONU [1] .

References

1 FAO/WHO/UNU, 2007. Protein and amino acid requirements in human nutrition: Report of a joint FAO/WHO/UNU expert consultation (WHO Technical Report Series 935). *Eds: World Health Organization,number of pages: 265/2007. ISBN: 92 4 120935 6.*
