# Supplementary material for: Essential oil optimizes the susceptibility of Callosobruchus maculatus and enhances the nutritional qualities of stored cowpea Vigna unguiculata
Source: R Soc Open Sci. 2017 Aug 23;4(8):170692. doi: 10.1098/rsos.170692 (PMC5579128; doi:10.1098/rsos.170692)
Supplement: Electronic supplementary materials (ESM 2) [file rsos170692supp2.pdf]

Electronic supplementary materials (ESM 2): Additional calculation formulas and experimental procedures

Essential oil optimizes the susceptibility of *Callosobruchus maculatus* and enhances the nutritional qualities of stored cowpea *Vigna unguiculata*

Mazarin Akami[1,2*], Hamada Chakira[2], Awawing A. Andongma[2], Kanjana Khaeso[2], Olajire A. Gbaye[3], Njintang Y. Nicolas[1,4], E-N Nukenine[1], and Chang-Ying Niu[2*]

Additional calculation formulas and experimental procedures

1. **Resistance Index of each generation as described by Olajire *et al*. [1] and modified as follows:**

$$\boldsymbol{R\ I = \frac{LCg}{LCs}}$$

Where:
$\boldsymbol{R\ I}$: Resistance Index;

$\boldsymbol{LCg}$: Lethal Concentration of each generation,

$\boldsymbol{LCs}$: Lethal Concentration of standard population.

2. **The Survival Rate (SR_g) of each products (EO and DDVP) at different generations:**

$$\boldsymbol{SR_g = \frac{\sum(\frac{20 - M_n}{20}) \times 100}{4}}$$

Where:
SR_g**:** Survival Rate per generation;

$\boldsymbol{n}$**:** Various concentrations used;

M_n: Mortality generated by each concentration per generation;

20: Total number of insects used;

4: Number of replications.

3. **The Chemical Score (CS)** was calculated as previously described by Block and Mitchell [2] and later reported by Elhardallou *et al.* [3]. It is defined as the ratio of a gram of the limiting amino acid in a tested sample **(AA_s)** to the same amount of the corresponding amino acid in a reference diet (whole-egg protein) **(AA_{ref})** multiplied by 100:

$$CS_i = \frac{AA_s i}{AA_{ref} i} \times 100$$

Where:

CS_i**:** Chemical Score of a given amino acid (***i***);

$AA_s i$**:** Amount of a given amino acid (***i***) in a sample;

$AA_{ref} i$: Corresponding amount of a given amino acid (***i***) in the reference sample.

4. **Essential amino acid index (EAAI)** was calculated according to Oser [4] with slight modifications. It is defined as a geometric mean of the ratios of the essential amino acids in the test protein (**EAAs**) relative to their corresponding amounts in the whole egg protein (**EAA_{ref}**).

$$EAAI_s = \frac{\sum\left(\frac{EAA_s i}{EAA_{ref} i}\right) \times 100}{N_{eaa} i}$$

Where:

$EAAI_s$**:** Essential Amino Acid Index of a given sample;

$EAA_s i$**:** Amount of given essential amino acid (***i***) in a sample;

$AA_{ref} i$: Amount of essential amino acid (***i***) in the reference sample;

$N_{eaa} i$**:** Number of a given amino acid (***i***) in a sample.

5. **The Net Protein Value (NPV)** was also evaluated as follows:

$$NPV = \frac{\text{Lowest amino acid score} \times \%\,\text{protein}}{100}$$

6. **The biological value (BV)** of cowpea seeds in each treatment was calculated using the method of Oser (1951)[4] as the follows:

$$\mathbf{BV = 1.09\ (EAA) - 11.7}$$

7. **Protein efficiency ratio (PER)** was estimated according to the regression equation reported by Alsmeyer *et al*. [5] and reported by Ilesanmi and Gungula [6]. Predicted protein efficiency ratio (P-PER) was computed as follows:

$$\mathbf{P\text{-}PER = -0.468 + 0.454\ (Leucine) - 0.105\ (Tyrosine)}$$

S2. Experimental designs

8. Total Carbohydrate determination

2g of cowpea flour was homogenized in 100ml of 95% ethanol. The homogenate was centrifuged at 1100 rpm for 10 min and used for the estimation of total sugar by anthrone method [7] as reported by Parthiban *et al.* [8].

Principle: Carbohydrate was first hydrolysed into simple sugars using dilute hydrochloric acid. In hot acidic medium, glucose is dehydrated to hydroxmethyl furfural. This compound forms with anthrone a green coloured product with absorption maximum at 630 nm.

Reagents:

- **Standard solution of glucose:** 100 mg of glucose was dissolved in 100 ml of water in a standard flask;
- **Working standard**: 10 ml of the stock was diluted to 100 ml. 1ml of this solution contains 100 µg of glucose;
- **Anthrone reagent:** 0.2% anthrone was dissolved in ice cold concentrated sulphuric acid. Prepared fresh before use;
- 2.5 N HCl.

S3. Experimental procedure

100mg of the sample was weighted and poured into a boiling tube, hydrolysed by keeping it in a boiling water bath for 3 hours with 5 ml of 2.5 N HCl and cooled to room temperature. It was neutralized with solid sodium carbonate until the effervescence ceases and the volume adjusted to 100 ml before centrifuging at 1000 rpm for 10 minutes. The supernatant was collected and 0.2 to 1 ml were taken for analysis. Prepare the standards by taking 0.2-1ml of the working standards. 1 ml of water serves as a blank made up the volume to 1ml in all the tubes with distilled water, and then added 4.0 ml of anthrone reagent, heated for 8 minutes in a boiling water bath, cooled rapidly and read the green to dark green colour at 630 nm using spectrophotometer (Eppendorf AG, Germany).

Calculation

A standard graph was drawn by taking the concentration of glucose on X axis and spectrophotometer reading on Y axis. From the graph the concentration of glucose in the sample was calculated.

9. Moisture Determination

5g of cowpea flour was placed in a crucible and dried at 110 ℃ to a constant weight after the initial weighing. Moisture content of the grains was calculated by the difference of initial and the final weight of the seed samples.

10. Ash Determination

5g of ground cowpea seeds was placed in a crucible, dried in oven at 100 ℃ and then burnt to ashes at 600 ℃ for 8hours in carbolite muffle furnace. This was cooled to room temperature until

a white ash was obtained. The ash content was determined by the difference in weight of the crucible before and after cooling.

11. Fat determination

Soxhlet ether extraction was used for fat extraction and quantification. Petroleum ether was added to 5g of cowpea seed flour and placed in an extraction apparatus (a thimble). Extraction was carried out for 18 hours. Soon after, ether was allowed to evaporate to dryness and only fat remained in the flask and was recovered. The amount of fat was determined by the difference in the weight of the flask before and after ether dryness[9].

12. Minerals determination

The total minerals of cowpea samples were determined using method No 970.12 [10-12]. Five grams of ash samples were dissolved in 10mL of 2M HNO_3 and oven-dried. An extra volume of 5 mL of 2M HNO_3 was then added, boiled and filtered through a Whatman filter paper into a 100ml volumetric flask and the filtrate was made up with distilled water. The Digital Flame Photometry (model PFP7) was used to determine the sodium, potassium and calcium. Total magnesium, iron, zinc, copper, sulfur and elements in traces (cobalt, boron, selenium, and manganese) were determined by atomic absorption spectrophotometer (BUCK 210VGP model) [10]. The Vanadomolybdate reagent at 400nm was used to determine and quantify Phosphorus from the sample filtrate by colorimetric method (Colorimeter SP 20, Bausch and Lamb).

References

1 Gbaye, O. A., Oyeniyi, E. A. & Ojo, O. B. 2016. Resistance of *Callosobruchus maculatus* (Fabricius)(Coleoptera: Bruchidae) Populations in Nigeria to Dichlorvos. *Jordan Journal of Biological Sciences* **9**.

2 Block, R. & Mitchell, H. in *Nutr. Abstr. Rev.* 249-278.

3 Elhardallou, S. B., Khalid, I. I., Gobouri, A. A. & Abdel-Hafez, S. H. 2015. Amino Acid Composition of Cowpea (Vigna ungiculata L. Walp) Flour and Its Protein Isolates. *Food and Nutrition Sciences* **6**, 790.

4 Oser, B. 1951. Method for integrating essential ammo acid content in the nutritional evaluation of protein. *Journal of the American Dietetic Association* **27**, 396-402.

5 Alsmeyer, R. H., Cunningham, A. & Happich, M. 1974. Equations predict PER from amino acid analysis. *Food Technology*.

6 Ilesanmi, J. & Gungula, D. 2016. Amino Acid Composition of Cowpea Grains Preserved With Mixtures OF Neem (Azadirachta indica) and Moringa (Moringa oleifera) Seed Oils. *American Journal of Food and Nutrition* **4**, 150-156. (doi:10.12691/ajfn-4-6-2).

7 Hodge, J. 1962. Determination of reducing sugars and carbohydrates. *Methods in carbohydrate chemistry* **1**, 380-394. (doi:10.1371/journal.pone.0065418).

8 Parthiban, V., Prakasam, V. & Prabakar, K. 2012. Changes in the biochemical constituents of carrot roots due to bacterial soft rot.

9 Teixeira, D. C., Farias, D. F., Carvalho, A. F. U., Arantes, M. R., Oliveira, J. T. A., Sousa, D. O. B., Pereira, M. L., Oliveira, H. D., Andrade-Neto, M. & Vasconcelos, I. M. 2013. Chemical composition, nutritive value, and toxicological evaluation of Bauhinia cheilantha seeds: a legume from semiarid regions widely used in folk medicine. *BioMed research international* **2013**.

10 Al-Maiman, S. A. & Ahmad, D. 2002. Changes in physical and chemical properties during pomegranate (Punica granatum L.) fruit maturation. *Food Chemistry* **76**, 437-441.

11 Ndidi, U. S., Ndidi, C. U., Olagunju, A., Muhammad, A., Billy, F. G. & Okpe, O. 2014. Proximate, antinutrients and mineral composition of raw and processed (boiled and roasted) Sphenostylis stenocarpa seeds from southern Kaduna, Northwest Nigeria. *ISRN nutrition* **2014**.

12 Olalekan, A. J. & Bosede, B. F. 2010. Comparative study on chemical composition and functional properties of three Nigerian legumes (jack beans, pigeon pea and cowpea). *Journal of Emerging Trends in Engineering and Applied Sciences* **1**, 89-95.
